# Realizing the Calculation of a Fully Normalized Associated Legendre Function Based on an FPGA

**DOI:** 10.3390/s24227262

**Published:** 2024-11-13

**Authors:** Yuxiang Fang, Qingbin Wang, Yichao Yang

**Affiliations:** School of Information and Electronic Engineering, Zhejiang University of Science and Technology, Hangzhou 310023, China; nbfangyuxiang66@163.com (Y.F.); yyc10140301@163.com (Y.Y.)

**Keywords:** associated Legendre function, gravity field, recursive algorithm, FPGA

## Abstract

A large number of fully normalized associated Legendre function (fnALF) calculations are required to compute Earth’s gravity field elements using ultra high-order gravity field coefficient models. In the surveying and mapping industry, researchers typically rely on CPU-based systems for these calculations, which leads to limitations in execution speed and power efficiency. Although modern CPUs improve instruction execution efficiency through instruction-level parallelism, the constraints of a shared memory architecture impose further limitations on the execution speed and power efficiency. This results in exponential increases in computation time as demand rises alongside high power consumption. In this article, we present a new computational implementation of an fnALF based on the ZYNQ platform. We design a task-parallel “pipeline” architecture which converts the original serial logic into a more efficient hardware implementation, and we utilize a redundant calculation layer to handle repetitive coefficient computations separately. The experimental results demonstrate that our system achieved accurate and rapid calculations. Under the only one geocentric residual latitude condition, we measured the computation times for spherical harmonic coefficient degrees of 360, 720, and 1080 to be 0.155922 s, 0.520950 s, and 1.401609 s, respectively. In the case of the multiple geocentric residual latitudes condition, our design generally yielded efficiency gains of over three times those of MATLAB R2020b implementation. Additionally, our calculated results were used to determine the geoid height in the field with an error of less than ±0.1m, confirming the reliability of our computations.

## 1. Introduction

Legendre functions have a wide range of applications in the precision orbiting of spacecraft, precise navigation of inertial navigation systems, inversion of the Earth’s gravity field, and calculations involving gravity field elements [1,2,3,4]. What needs to be used in these practical engineering application scenarios is often a normalized Legendre function, which we refer to as a fully normalized associated Legendre function (fnALF). With the increasing demands of sensor systems, achieving higher-order fnALFs for fast and accurate computation of sensor-driven functions has become an essential challenge. In order to obtain a higher-order, fully normalized aggregated Legendre function through a lower-order, fully normalized aggregated Legendre function, researchers usually calculate it recursively by using the properties of the aggregated Legendre function. The authors of [5] proposed a standard forward-column recursive form of a fully normalized aggregated Legendre function as a way of estimating spherical harmonic coefficients from spherical sampling data. The authors of [6] proposed computation of the non-normalized spherical harmonic function by improving the coefficients and then transforming the computation into the fnALFs. This new fast formula is known as the Belikov recursive formula. The authors of [7] proposed a recursive method between every other order and degree formulation, and it was suitable for recursive computation between aggregated Legendre functions of different orders with high computational efficiency and accuracy. Therefore, this algorithm is often used for the computation of ultra high-order, fully normalized aggregated Legendre function. From the above, we can learn that the recursive calculation of the solution for the cohesive Legendre function through the properties of the function can be categorized into three methods: the recursive method according to the degree (order), the Belikov recursive method, and the recursive method between every other order and degree.

The computation of these fnALFs using a recursive formulas has traditionally been performed on computers using programming languages such as C++ and Fortran and software platforms such as MATLAB. However, this has some limitations:

- Modern computers can execute multiple instructions simultaneously within a single processor core and utilize multiple cores to improve computing performance, but they still have limitations when handling highly complex or resource-intensive tasks. This is because traditional CPUs primarily rely on time-sharing and task-switching mechanisms, which can lead to inefficiencies in achieving true parallelism. Moreover, as the computation order increases, the exponential growth in computational complexity and memory access bottlenecks can hinder performance. These limitations make it challenging for conventional computers to meet the fast requirements of high-order fnALF calculations.
- This is because of the high power consumption required to run the formula on a computer in the traditional way. For example, we use desktop processors such as Intel Core i5 and i7 (Intel Corporation, Santa Clara, CA, USA) and AMD Ryzen 5 and 7 (AMD, Santa Clara, CA, USA) to perform computing tasks, which typically consume between 65 W and 125 W. Even low-power processors typically consume between 15 W and 35 W. In some specific engineering environments, it is not possible to fulfill such high power requirements.
- In geology, when the experimental environment is outdoors, for real-time analysis of the calculation results, the calculation equipment often needs to be miniaturized and convenient. However, the traditional approach cannot meet this kind of demand, which means that the secondary calculation and analysis of the collected data will be delayed.

In this study, after investigating the limitations that exist in traditional computational approaches, we propose an FPGA-based solution to realize the computation of fnALFs. This solution is adapted to an FPGA hardware platform by parallelizing the computation of the recursive formulation of fnALFs. In addition, we optimize the computational terms in the formula to increase the speed of computation and reduce the consumption of resources. Finally, the requirements of high efficiency, ease of use, and low power consumption for the calculation of fnALFs are realized.

Researchers both domestic and international have made significant advances in improving computational efficiency to reduce power consumption using FPGAs. For instance, the authors of [8] designed a tsunami propagation code based on the MOST algorithm to predict the time required for the arrival of a tsunami, and in order to be able to implement high-performance computing on an FPGA, the authors wrote a specific Opencl kernel for the implementation, which achieved a notably good computational speedup. The authors of [9] optimized the traditional SampEn algorithm for the limitations of computational complexity and real-time applications by accelerating the SampEn algorithm with FPGAs and utilizing a reasonable scheduling strategy to deal with the load imbalance problem. They achieved acceleration results of more than two orders of magnitude. The authors of [10] compared schemes for implementing iteration and recursion in FPGA circuits. The computation of fnALFs is essentially an iterative computation, and thus this provides us with ideas for designing an implementation. The authors of [11] reduced the presence ratio by means of precomputed parameters in order to run a binarized neural network (BNN) more efficiently on edge devices. The computational formula was simplified using the model inference feature, which achieved a speedup in computation without affecting the accuracy.

There has also been a great deal of research and analysis in search of ways of computing ultra high-degree and ultra high-order fully normalized associated Legendre functions. The authors of [12] compared and analyzed commonly used methods for ultra high-degree and ultra high-order fully normalized associated Legendre functions by means of computation on a computer and proved that the recursive method between every other order and degree is a better method for calculating ultra high-degree and ultra high-order fully normalized associated Legendre functions. The authors of [13] compared the advantages and disadvantages of various methods for calculating ultra high-degree and ultra high-order fully normalized associated Legendre functions and came to the same conclusions as those in [12]. The authors of [14] summarized various common methods for computing a fully normalized associated Legendre function and its derivatives, and they were tested numerically. The results of the tests showed that the algorithms which used a recursive method between every other order and degree had good stability.

Collectively, the research on improving computational efficiency through parallel processing on FPGAs, as well as the comparison and analysis of ultra high-degree and ultra high-order fully normalized associated Legendre functions, provided a significant reference for this study’s implementation of fully normalized associated Legendre functions on an FPGAs and greatly contributed to the design of this work.

## 2. Related Work

### 2.1. Theoretical Analysis of Fully Normalized Associated Legendre Functions

In order to obtain a fully normalized aggregated Legendre function, we need a Legendre differential equation [15]. The expression of Legendre’s differential equation is shown in Equation (1):

(1) $$\left(1-x^{2}\right)y^{\prime \prime }\left(x\right)-2xy^{\prime }\left(x\right)+n\left(n+1\right)y\left(x\right)=0$$

We need to take the *k*th derivative of *x* in Equation (1). Then, we obtain a new equation (Equation (2)):

(2) $${\left[\left(1-x^{2}\right)y^{\prime \prime }\left(x\right)\right]}^{k}-2{\left[xy^{\prime }\left(x\right)\right]}^{k}+n\left(n+1\right){\left[y\left(x\right)\right]}^{k}=0$$

For Equation (2), we expand the Leibniz criterion with the first term and the second application. The first and second terms after the expansion are

(3) $${\left[\left(1-x^{2}\right)y^{\prime \prime }\left(x\right)\right]}^{k}=\left(1-x^{2}\right){\left[y\left(x\right)\right]}^{\left(k+2\right)}-2kx{\left[y\left(x\right)\right]}^{\left(k+1\right)}-k\left(k-1\right){\left[y\left(x\right)\right]}^{\left(k\right)}$$

(4) $${\left[xy^{\prime }\left(x\right)\right]}^{k}=x{\left[y\left(x\right)\right]}^{k+1}+k{\left[y\left(x\right)\right]}^{k}$$

Substituting Equations (3) and (4) into Equation (2) and organizing them yields

(5) $$\left(1-x^{2}\right){\left[y\left(x\right)\right]}^{k+2}-2\left(k+1\right)x{\left[y\left(x\right)\right]}^{\left(k+1\right)}+\left[n\left(n+1\right)-k\left(k+1\right)\right]{\left[y\left(x\right)\right]}^{\left(k\right)}=0$$

To further simplify Equation (5), we need to set up a new equation [16]:

(6) $${\left[y(x)\right]}^{\left(k\right)}=u\left(x\right){\left(1-x^{2}\right)}^{-\frac{k}{2}}$$

With Equation (5), we can easily solve for ${\left[y\left(x\right)\right]}^{k+1}$ and ${\left[y\left(x\right)\right]}^{k+2}$. By substituting these results into the band in Equation (5), we obtain Equation (7):

(7) $$\left(1-x^{2}\right)u^{\prime \prime }\left(x\right)-2xu^{\prime }\left(x\right)+\left[n\left(n+1\right)-\frac{k^{2}}{1-k^{2}}\right]u\left(x\right)=0$$

If *k* = 0 in Equation (7), then we obtain the original Equation (1). Thus, we will call Equation (7) the aggregated Legendre differential function.

Using Equation (6), we can solve for the solution u(x):

(8) $$u\left(x\right)={\left(1-x^{2}\right)}^{\frac{k}{2}}\frac{d^{k}y\left(x\right)}{dx^{k}}$$

Here, the solution to the equation u(x) is the solution to the summed Legendre differential equation, which is usually denoted as $P_{nm}$:

(9) $$P_{nm}\left(x\right)={\left(1-x^{2}\right)}^{\frac{m}{2}}\frac{d^{m}P_{n}\left(x\right)}{dx^{m}}$$

where *n* is called the spherical harmonic coefficient degree (degree), *m* is called the spherical harmonic coefficient order (order), and *x* is in the zone of −1<x<1.

If the calculation is for a sphere, then *x* needs to be transformed into cos(θ):

(10) $$P_{nm}\left(cos\left(\theta \right)\right)=\sin^{m}\theta \frac{d^{m}P_{n}\left(cos\left(\theta \right)\right)}{d{\left(cos\theta \right)}^{m}}$$

We can derive the solution from the recurrence relation in Equation (10):

(11) $$\left\{\begin{array}{c} P_{1,1}\left(cos\theta \right)=sin\theta \\ P_{2,1}\left(cos\theta \right)=3cos\theta sin\theta \\ P_{2,2}\left(cos\theta \right)=3sin^{2}\theta \\ \cdots \end{array}\right.$$

This kind of definition can be used to solve low-order aggregated Legendre functions quickly and easily. However, if a high order is needed, then this will be extremely troublesome to calculate by definition. The solution $P_{nm}$ will also have a long expression, which is not favorable for practical applications. Therefore, recursive formulas are needed to simplify the process.

### 2.2. Theory of the Recursive Method Between Every Other Order and Degree

The recursive calculation of the solution for a cohesive Legendre function through its properties can be categorized into three approaches: the recursive method according to the degree (order), the Belikov recursive method, and the recursive method between every other order and degree. In this study’s FPGA design and implementation of the recursive formulation of fnALFs, we chose to use the recursive method between every other order and degree. This was because the recursive method between every other order and degree had high computational accuracy. The formulas for recursive computation change when different order numbers are selected:

(12) $$\left\{\begin{array}{cc} {\bar{P}}_{nm}\left(\cos\theta \right)=a_{nm}t{\bar{P}}_{n-1,m}\left(\cos\theta \right)-b_{nm}{\bar{P}}_{n-2,m}\left(\cos\theta \right) & m=0,1\\ {\bar{P}}_{nm}\left(\cos\theta \right)=\alpha_{nm}{\bar{P}}_{n-2,m}\left(\cos\theta \right)-\beta_{nm}{\bar{P}}_{n-2,m-2}\left(\cos\theta \right)-\gamma_{nm}{\bar{P}}_{n,m-2}\left(\cos\theta \right) & m\geq 2 \end{array}\right.$$

Here, t=cosθ. The coefficients $a_{nm}$, $b_{nm}$, $\alpha_{nm}$, $\beta_{nm}$, and $\gamma_{nm}$ need to be calculated first. The formula for calculating the coefficients is presented in Equation (12):

(13) $$a_{nm}=\sqrt{\frac{\left(2n-1)(2n+1\right)}{\left(n-m)(n+m\right)}}$$

(14) $$b_{nm}=\sqrt{\frac{\left(2n+1)(n+m-1)(n-m-1\right)}{\left(n-m)(n+m)(2n-3\right)}}$$

(15) $$\alpha_{nm}=\sqrt{\frac{\left(2n+1)(n-m)(n-m-1\right)}{\left(2n-3)(n+m)(n+m-1\right)}}$$

(16) $$\beta_{nm}=\sqrt{K}\sqrt{\frac{\left(2n+1)(n-m)(n+m-3\right)}{\left(2n-3)(n+m)(n+m-1\right)}},$$

$$\left\{\begin{array}{c} K=2,m=2\\ K=1,m>2 \end{array}\right.$$

(17) $$\gamma_{nm}=\sqrt{\frac{\left(n-m+1)(n-m+2\right)}{\left(n+m)(n+m-1\right)}}$$

**Figure 1:**
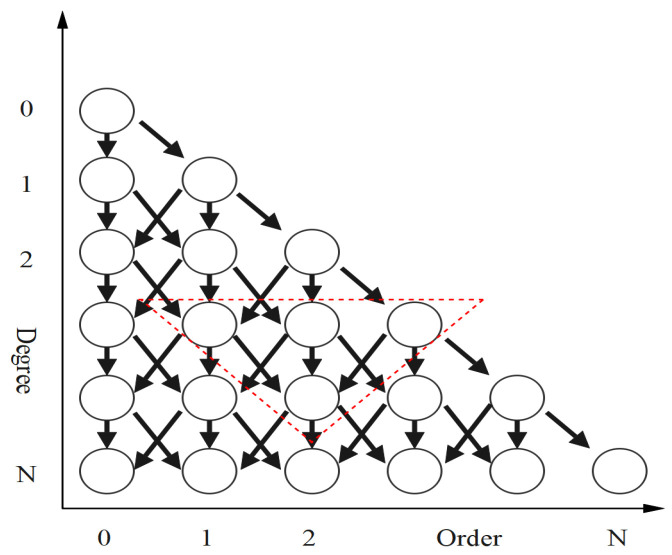
The recursive process. The dashed triangle means a set of numbers.

When m>2, the values of the recursion coefficients are all less than one, and thus the algorithm for the recursive order at every other degree is stable and reliable. In Figure 1, we give the procedure of the recursive method between every other order and degree. Each combination of n and m in Equation (12) corresponds to each black circle in the figure, and one can see the relationship between each combination computed. In Algorithm 1, we give the pseudocode for implementation of the recursive method between every other order and degree, showing in detail how the recursive method between every other order and degree can be used to computationally find the fnALF.
**Algorithm 1:** Implementation of the recursive algorithm for every other order and degree.**Input:** 
θ, *N*, Leg[1][0], Leg[1][0]**Output:** 
Leg[n][m]  1:Initialization values  2:**for** n=2 **to** *N* **do**  3:   **for** m=0 **to** *n* **do**  4:     **if** m<2 **then**  5:        Calculation of scaling factors a,b;  6:        Update Leg[n][m]; m=2  7:     **else if** m=2 **then**  8:        K=1;  9:        Calculation of scaling factors α,β,γ;10:        Update Leg[n][m];11:     **else**12:        K=2;13:        Updated calculation of scaling factors α,β,γ;14:        Update Leg[n][m];15:     **end if**16:   **end for**17:**end for**18:**return** Leg[n][m]

## 3. FPGA-Based Hardware and Software Architecture Design

### 3.1. Overall Structural Plan

**Figure 2:**
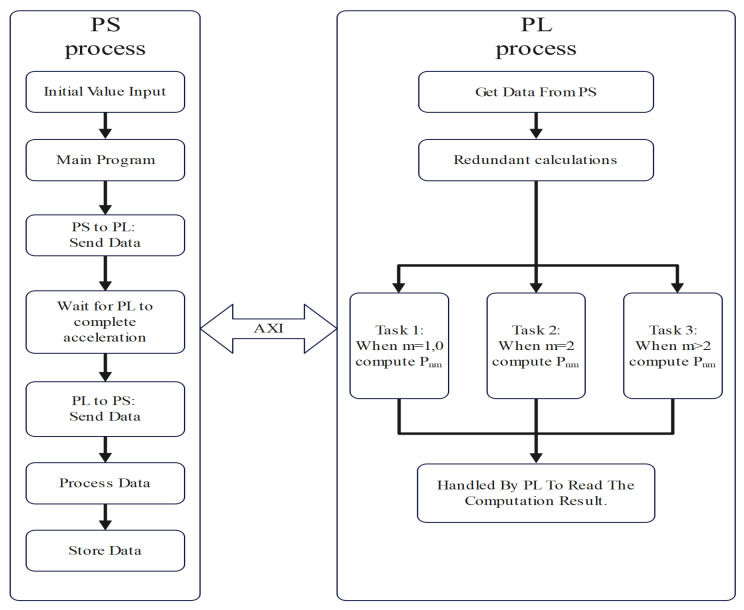
Basic architecture design.

This study was based on the Vivado 2021.2 development environment on an AX7020 development board using Xilinx’s XC7Z020-2CLG484I (Xilinx, San Jose, CA, USA) as the FPGA master chip. The number of computationally relevant intermediates grew exponentially as the order of the fnALF increased. Therefore, it was unrealistic to implement all computations and storage on a pure FPGA. We considered the resource limitation issue. It is well known that the Zynq-7020 core fuses programmable logic (PL) with a high-performance ARM processor core (PS), and thus it was possible to fully utilize the parallel processing power and highly programmable nature of the PL part of the FPGA to enhance the computational efficiency, and the PS was utilized for the rest of the operations. As shown in Figure 2, we implemented our algorithm in two parts based on the properties of ZYNQ.

In the beginning, the PS side received the initial value which we set for the calculation and sent it over the AXI bus to the PL side for calculation. The calculation result was then sent back to the PS side through the AXI bus. In the computation module, we adopted the pipeline design principle of independent stage processing. We divided our algorithm into three independent tasks based on the if-else–if-else hierarchy. The judgment conditions from the original hierarchical relationship were no longer considered in the specific calculations, allowing the three independent tasks to be executed in parallel. The final processing of the settlement results was delegated to the PS side for further handling. The specific design method will be explained in the following introduction to the calculation module.

### 3.2. Reduction in Arithmetic Operations

The computational module is the key to improving the computational efficiency, and thus we specially designed a computational module for the recursive method between every other order and degree and the characteristics of the hardware platform. As can be seen in Algorithm 1, if the conventional serial programming method were to be used, then the calculation of fnALFs would require sequential judgment in the order of the spherical harmonic coefficients. However, in the last case, there is a waste of time by waiting unnecessarily because the first two cases are still judged to be waiting for the execution to be completed. However, in this case, we do not need the results of the calculations in the first two cases. Therefore, we need a new computational approach to solve such problems which originally occurred with serial computation. In this study, we split the computation of the recursive method between every other order and degree according to the three judgments after the different spherical harmonic coefficients into three separate computational modules. These three independent computational modules worked together to perform computational processing tasks. For the processing of the calculation results, we added a signal “type” to the three independent calculation modules to select the category of the calculation results. This category selection signal was handed over to the PS side to select the spherical harmonic coefficient order. In this way, the original serial computation method was replaced by a parallel computation method which was more suitable for FPGA computation.

In the actual calculation, we found that there were some values which needed to be repeated in each calculation of coefficients. If we would repeat these values in every calculation, then this would greatly affect the computational efficiency and resources. For this reason, we simplified the calculation of coefficients in Equations (13)–(15) for the recursive method between every other order and degree as follows:

(18) $$a_{nm}=\sqrt{\frac{\left(i-1)(i+1\right)}{uv}}$$

(19) $$b_{nm}=\sqrt{\frac{\left(i+1)(v-1)(u-1\right)}{uv(i-3)}}$$

(20) $$\alpha_{nm}=\sqrt{\frac{u(i+1)(u-1)}{v(i-3)(v-1)}}$$

(21) $$\beta_{nm}=\sqrt{K}\sqrt{\frac{u(i+1)(v-3)}{v(i-3)(v-1)}},$$

$$\left\{\begin{array}{c} K=2,m=2\\ K=1,m>2 \end{array}\right.$$

(22) $$\gamma_{nm}=\sqrt{\frac{\left(u+1)(u+2\right)}{v(v-1)}}$$

**Figure 3:**
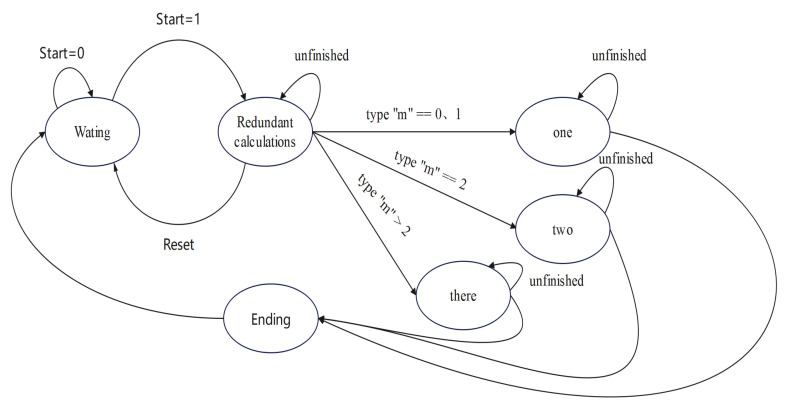
A processing diagram of the optimized recursive method between every other order and degree.

In Equations (13)–(17), we calculate anm by performing several calculations on the terms 2n, n−m, and n+m to obtain their results. Therefore, we can simplify the formula in Equations (18)–(22). We simplify multiple operations into calculations with fewer parameters. We replace 2n, n−m, and n+m in the original formula with *i*, *u*, and *v*, respectively. We opened a new layer to compute these substituted values. We named this layer “redundant calculations”. The results of this layer were given directly for the subsequent calculations, thus avoiding the time and resource consumption caused by repeated calculations. Figure 3 shows the way that we implemented the recursive method between every other order and degree after introducing the new layer.

The whole computational module was idle after power-up, and when the initial value reached the new layer added above, it was subjected to redundant calculations. After the redundant calculations were completed, the three calculation tasks corresponding to circles 1, 3, and 4 in Figure 3 began. When the three computational tasks were completed, the “type” information was carried to the subsequent PS side. The PS side then selected the correct calculation results. This operation was repeated until we calculated the degree that we needed.

## 4. Simulation Debugging

### 4.1. Algorithm Accuracy Analysis

In order to verify the effectiveness of the above algorithm, we utilized the serial computation algorithm in the Visual Studio 2022 software environment for different spherical harmonic coefficient orders and levels. Because fnALFs have a large number of point locations (combinations of n and m) at higher orders, we selected some of them for presentation in Table 1. At the same time, we also show the results of calculating the corresponding points on the FPGA.

In this study, in addition to comparing the accuracy of individual values, a comparative accuracy analysis of the overall error was performed. In the overall accuracy analysis, we eliminated missing values caused by exceeding the range of single-precision floating-point numbers. Table 2 shows the statistics of the root mean square error and mean absolute error at each spherical harmonic coefficient degree.

From Table 1 and Table 2, it can be seen that the difference between the computational implementation designed in this study and the results obtained from the computation in Visual Studio 2022 was not significant at low spherical harmonic coefficient degrees. However, the accuracy of the computation decreased as the spherical harmonic coefficient degree increased, which may have been due to errors in the lookup table and division calculations based on the FPGA platform. The overall error was not large, which indicated that the computational accuracy met the expectations and verified the accuracy of the design in this study.

### 4.2. Simulated Waveforms for the Recurrence Coefficient

**Figure 4:**
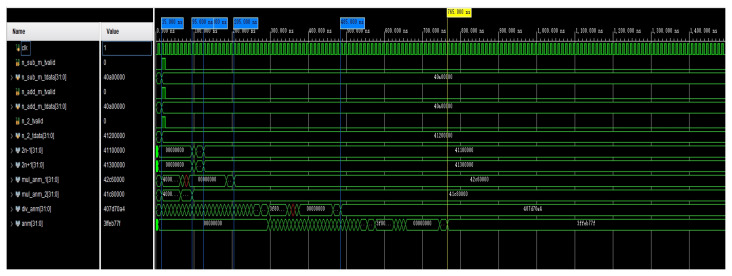
A simulation of the calculation of the waveform of $a_{nm}$.

An algorithm was designed for waveform simulation on an FPGA platform. A 100 MHz simulation clock was employed in the testbench. In the introduction to this study, we mentioned that there are many combinations of *n* and *m*. Out of the many combinations of *n* and *m*, we chose n=5 and m=0 for the experiments with the simulation of $a_{nm}$ and $b_{nm}$. The simulation of the computed waveform of the coefficient $a_{nm}$ is shown in Figure 4. In the optimized calculation, n_sub_m_tdata, n_add_m_tdata, and n_2_tdata were used as the initial input values for the calculation. The signals named in the figure beginning with mul and div are multiplication and division operations on $a_{nm}$, respectively, and they include the calculation of two intermediate quantities. The waveform simulation results show that when using two adders or subtractors, the operation was completed in 125 ns. Once these intermediate quantities were computed, the computation of $a_{nm}$ began and was completed in 765 ns. This completed the calculation of Equation (18). In this way, parallel computation on the FPGA took 750 ns, and if full serial computation was used, then it took 940 ns.

**Figure 5:**
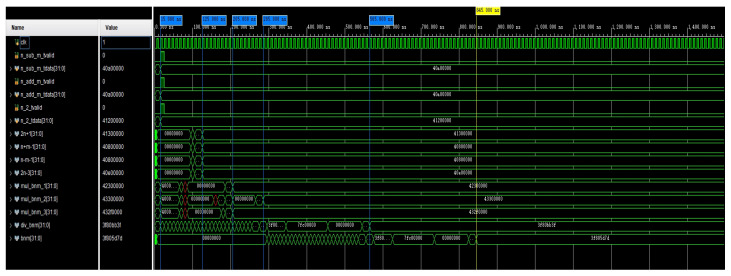
A simulation of the calculation of the waveform of $b_{nm}$.

The simulation of the computed waveform of the coefficient $b_{nm}$ is shown in Figure 5. In the optimized calculation, n_sub_m_tdata, n_add_m_tdata, and n_2_tdata were used as the initial input values for the calculation. The signals named in the figure beginning with mul and div are multiplication and division operations on $b_{nm}$, respectively, and they include the calculation of four intermediate quantities. The waveform simulation results show that when using four adders or subtractors, the operation was completed in 125 ns. Once the intermediate quantities were computed, the computation of $b_{nm}$ began and was completed in 845 ns. This completed the calculation of Equation (19). In this way, parallel computation on the FPGA took 830 ns, and if full serial computation was used, then it took 1240 ns.

Coefficients α, β, and γ were used when the thermal harmonic coefficient order was greater than or equal to two. Thus, for the simulated waveform experiments on α, β, and γ, *n* = 5 and *m* = 2 were chosen as the combination of *n* and *m*.

**Figure 6:**
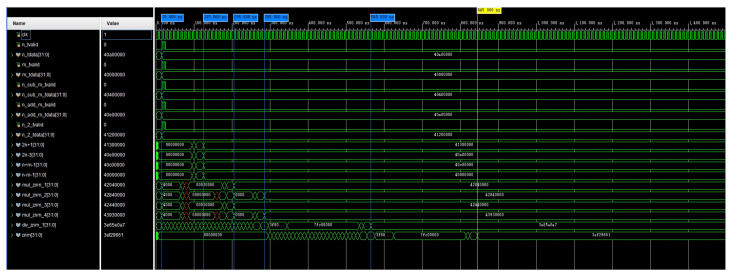
A simulation of the calculation of the waveform of $\alpha_{nm}$.

The simulation of the computed waveform of the coefficient $\alpha_{nm}$ is shown in Figure 6. In the optimized calculation, n_sub_m_tdata, n_add_m_tdata, and n_2_tdata were used as the initial input values for the calculation. In the figure, $\alpha_{nm}$ is named $z_{nm}$. The signals named in the figure beginning with mul and div are multiplication and division operations of $\alpha_{nm}$, and they include the calculation of four intermediate quantities. The waveform simulation results show that when using four adders or subtractors, the operation was completed in 125 ns. Once the intermediate quantities were computed, the computation of $\alpha_{nm}$ began and was completed in 845 ns. This completed the calculation of Equation (20). In this way, parallel computation on the FPGA consumed 830 ns, and if full serial computation was used, then it took 1320 ns.

**Figure 7:**
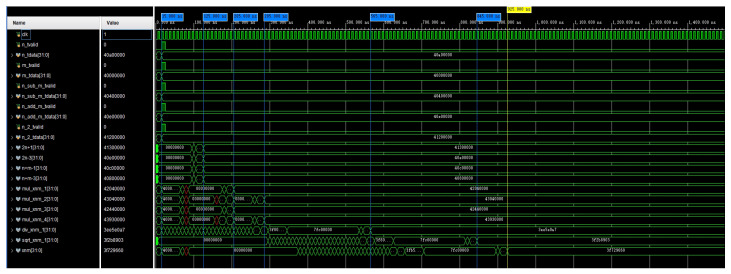
A simulation of the calculation of the waveform of $\beta_{nm}$.

The simulation of the computed waveform of the coefficient $\beta_{nm}$ is shown in Figure 7. In the optimized calculation, n_sub_m_tdata, n_add_m_tdata, and n_2_tdata were used as the initial input values for the calculation. In the figure, $\beta_{nm}$ is named $x_{nm}$. The signals named in the figure beginning with mul and div are multiplication and division operations of $\beta_{nm}$, respectively, and they include the calculation of four intermediate quantities. The waveform simulation results show that when using four adders or subtractors, the operation was completed in 125 ns. Once these intermediate quantities were computed, the computation of $\beta_{nm}$ began and was completed in 925 ns. This completed the calculation of Equation (21). In this way, parallel computation on the FPGA took 905 ns, and if full serial computation was used, then it took 1400 ns.

**Figure 8:**
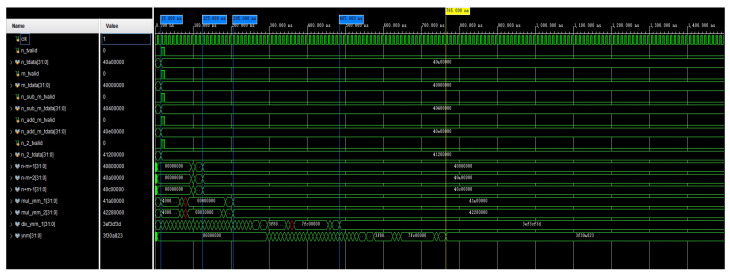
A simulation of the calculation of the waveform of $\gamma_{nm}$.

The simulation of the computed waveform of the coefficient $\gamma_{nm}$ is shown in Figure 8. In the optimized calculation, n_sub_m_tdata, n_add_m_tdata, and n_2_tdata were used as the input initial values for the calculation. In the figure, $\gamma_{nm}$ is named $y_{nm}$. The signals named in the figure beginning with mul and div are multiplication and division operations on $\gamma_{nm}$, respectively, and they include the calculation of three intermediate quantities. The waveform simulation results show that when using three adders or subtractors, the operation was completed in 125 ns. Once the intermediate quantities were computed, the computation of $\gamma_{nm}$ began and was completed in 765 ns. This completed the calculation of Equation (22). In this way, parallel computation on the FPGA took 750 ns, and if full serial computation was used, then it took 1050 ns.

**Figure 9:**
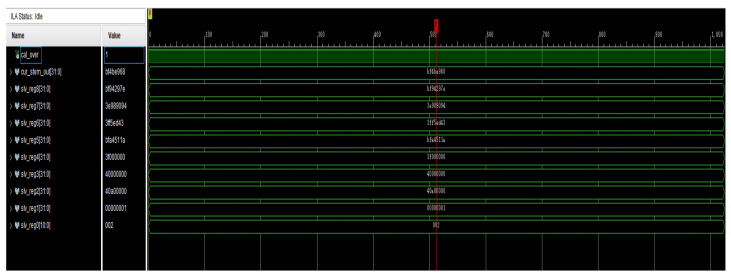
Data interaction via AXI4-Lite.

Figure 9 shows the content of the data interaction between the PS and PL via AXI4-Lite. In the figure, slv_reg0–slv_reg8 correspond to the function type, control start calculation, *i*, *m*, sin(θ), $P_{n-1,m}$, $P_{n-2,m}$, $P_{n,m-2}$, and $P_{n-2,m-2}$, respectively.

In Figure 9, we calculated m=2, and type 002 was correctly selected. The correctness of our design in Figure 3 is verified.

### 4.3. Time Consumption Analysis

As shown in Table 3, we carried out single-point experimental calculations for the recursive algorithm using every other order and degree for a geocentric residual latitude of 60° at spherical harmonic coefficient degrees of 360, 720, and 1080. In the experiment, our FPGA used a clock frequency of 200 Mhz provided by the PS side.

**Figure 10:**
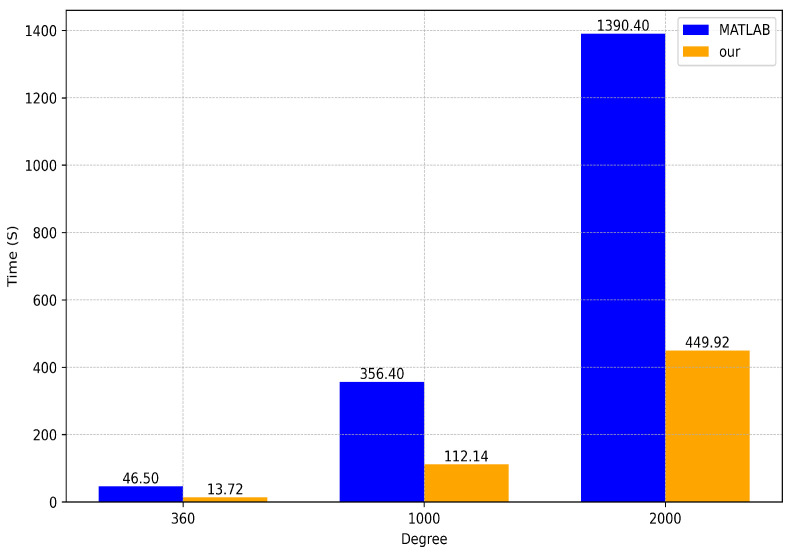
Performance comparison according to time consumption.

We conducted additional experiments to eliminate the effect of the chosen geocentric residual latitude on the calculations. We selected geocentric residual latitudes from 1 to 89 with an interval of one for a total of 90 standard points, and then we computed their time consumption for different spherical harmonic coefficient orders for comparison with those computed with MATLAB [17]. The parameters are shown in Figure 10.

The FPGA-based implementation of the cross-order recursive algorithm in our design had a significant advantage in terms of time consumption over the implementation on the MATLAB platform using matrix operations. When *n* = 360, our computational efficiency was 3.38× higher than when using MATLAB. When *n* = 1000, our computational efficiency was 3.17× higher than that when using MATLAB. When *n* = 2000, our computational efficiency was 3.09× higher than when using MATLAB. The average computational efficiency improvement when using the FPGA rather than MATLAB was more than threefold higher.

### 4.4. Power Consumption Evaluation

The number of hardware resources for the FPGA-based recursive method between every other order and degree designed in this study is presented in Table 4. In this design, DSPs were utilized for multiplication, and LUTs were utilized for addition, as the operations involved floating-point numbers and required efficient computation. Although only 49 DSPs were employed, this was sufficient due to optimization strategies which reduced the need for additional resources. Furthermore, there remains flexibility in resource allocation for future modifications. As shown in Table 4, the hardware resources operated within a reasonable range, balancing performance without overloading the system and effectively meeting the hardware acceleration requirements.

**Figure 11:**
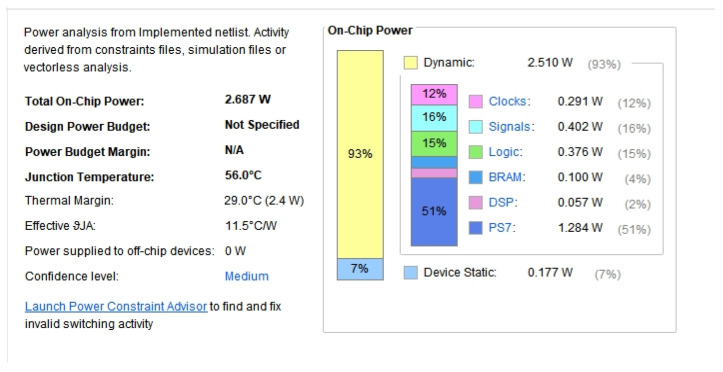
Calculated power consumption.

According to the power consumption analysis using Vivado 2021.2, as shown in Figure 11, the total on-chip power consumption of this design was estimated to be 2.687 W, while our actual measurement was 2.9 W, both of which are significantly lower than the power consumption of using a computer.

### 4.5. FPGA Implementation of Geoid Height

**Figure 12:**
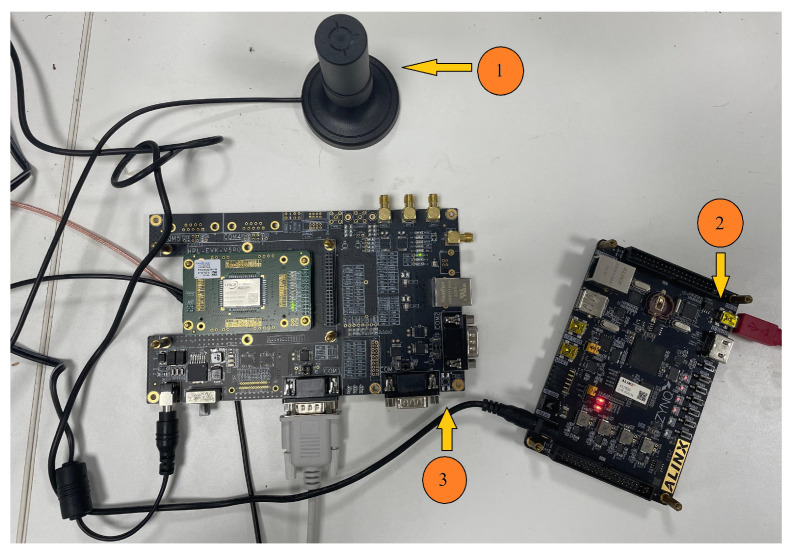
Experimental platform for calculation of the geoid height with the FPGA implementation. (1) Receiving antenna. (2) ZYNQ-7020. (3) UM980.

**Figure 13:**
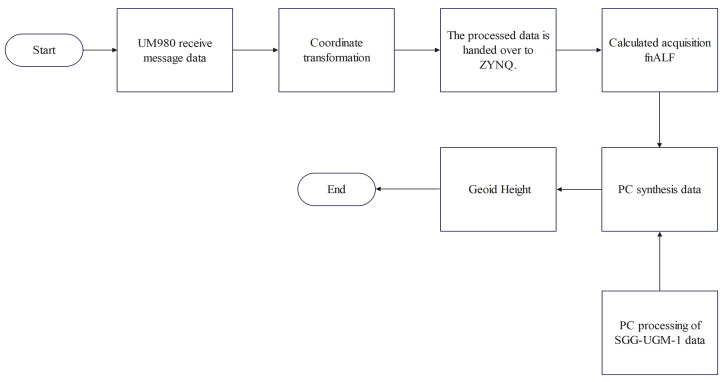
Flowchart for calculation of geoid height.

Our optimization of the recursive method between every other order and degree was coded and implemented in Verilog, synthesized using the Xilinx Vitis 2021.2, and implemented in ZYNQ-7020 (XC7Z020-2CLG400). In order to calculate the geoid height in the Earth’s gravitational field, we performed field experiments. The gravitational field model used in this experiment was SGG-UGM-1, and the rest of the required computational quantities were obtained by means of a UM980 (Unicorecom, Beijing, China) compass sensor. The SGG-UGM-1 model which we used is available on the ICGEM website. Table 5 shows the correlation coefficients for the observation accuracy of the UM980 module. Table 6 shows the geoid height according to the UM980 acceptance and the geoid height calculated with our system. Figure 12 shows the experimental platform. Figure 13 shows the flow of data processing.

To calculate the geoid height in the Earth’s gravitational field, we needed to obtain the longitude and latitude coordinates of the experimental points from UM980. However, the original latitude and longitude coordinates obtained cannot be directly used for calculation; they need to undergo the coordinate transformation process in Figure 13 to obtain the new coordinate information. The new coordinate information was used to provide the initial values for calculating the required fnALF values in ZYNQ. Subsequently, we obtained the relevant coefficients for each degree from the SGG-UGM-1 model and combined both to derive the geoid height.

As can be seen in Table 6, the difference between the received geoid height and the calculated geoid height was ±0.1m. This error indicates that our system can basically meet the needs of outdoor real-time calculation operations.

## 5. Conclusions

In this study, we designed an FPGA-based design for a recursive method between every other order and degree for fnALFs. The designed implementation solved the problem of high time consumption for computation which was originally brought about by the serial computation method. The proposed hardware design takes the problem of repeated computation of values encountered in the cross-order algorithm due to the computation of coefficients into account and provides a solution for the de-redundancy of repeated computations, thus reducing the unnecessary wasting of resources.

We took advantage of the parallel computing of an FPGA and used pipelining ideas to improve the speed of coefficient computation and the calculation when summing Legendre functions. This design utilized an FPGA to reduce the size and power consumption for the calculation of fnALFs, thus getting rid of the dependence of existing algorithms on computers and significantly reducing the calculation time.

The design was implemented in a ZYNQ FPGA device from Xilinx, and the computational modules of the design were simulated using the Vivado Simulator in Vivado2021.2. The simulation results show that the designed system was able to accurately realize the expected parallel computation. The implementation in this study had a higher computational efficiency than that of the traditional serial implementation. A comparison between the accuracy obtained in this study and that of computed values obtained from a computer showed that the scheme implemented in this study had good computational accuracy when using single-precision floating-point numbers. The computational efficiency was much higher than that of MATLAB’s matrix computation method in the case of multiple geocentric residual latitudes.

Overall, the FPGA-based recursive method between every other order and degree achieved better performance. The designed function calculation system can be used in the study of Earth’s gravitation field, which gives it broad application prospects.

## Figures and Tables

**Table 1:** Calculation of fnALF points.

Number	(n,m)	Calculation by the FPGA	Calculation by the PC
1	(3, 0)	−1.15751	−1.15752
2	(3, 2)	1.9213	1.9213
3	(100, 0)	−0.8579898	−0.857991
4	(100, 99)	$2.162901\times 10^{-5}$	$2.20075\times 10^{-5}$
5	(200, 0)	−0.3134005	−0.313402
6	(200, 199)	$2.094115\times 10^{-11}$	$2.09412\times 10^{-11}$
7	(360, 0)	1.171138	1.17114
8	(360, 359)	$3.289839\times 10^{-21}$	$3.28986\times 10^{-21}$
9	(720, 0)	1.171170	1.17118
10	(720, 719)	–	$1.79417\times 10^{-43}$
11	(1080, 0)	1.171181	1.17119
12	(1080, 1079)	–	$7.88719\times 10^{-66}$

Some of the values are missing from the table because the calculated values were outside of the range of the single-precision floating-point numbers we used.

**Table 2:** Calculation error statistics at each spherical harmonic coefficient degree.

Spherical Harmonic Coefficient Degree	RMSE	MAE
360	0.000745993	0.000000256663
720	0.002174622	0.000001729976
1080	0.004569649	0.000003829346

**Table 3:** The real execution times for a geocentric residual latitude of 60° with spherical harmonic coefficient degrees of 360, 720, and 1080.

Spherical Harmonic Coefficient Degree	Time (s)
360	0.155922
720	0.620950
1080	1.401609

**Table 4:** Calculation of the hardware resources to be consumed.

Resource	Utilization	Available	Utilization (%)
LUT	23,732	53,200	44.61
FF	43,495	106,400	40.88
BRAM	39.5	140	28.21
DSP	49	220	22.27

**Table 5:** Correlation coefficient for observation accuracy when using UM980.

Signal	BDS	GPS	GLONASS	GALILEO
B1I/B1C/L1C/L1C/A/G1/E1				
Pseudo range	10 cm	10 cm	10 cm	10 cm
B1I/B1C/L1C/L1C/A/G1/E1				
Carrier phase	1 mm	1 mm	1 mm	1 mm
B2I/B2a/B2b/L5/E5a/E5b				
Pseudo range	10 cm	10 cm	10 cm	10 cm
B2I/B2a/B2b/L5/E5a/E5b				
Carrier phase	1 mm	1 mm	1 mm	1 mm
B3I/L2P(Y)/L2C/G2				
Pseudo range	10 cm	10 cm	10 cm	10 cm
B3I/L2P(Y)/L2C/G2				
Carrier phase	1 mm	1 mm	1 mm	1 mm

**Table 6:** Calculation of the geoid height and the received geoid height.

Methodology	East Longitude, South Latitude	Geoid Height (m)
Our system	120.023, 30.224	7.93142467
Receivers	120.02393481092, 30.22446254192	7.8484

## Data Availability

The data were described in detail in the explanation of the experimental set-up and can be made available upon request.
